# Association Between Coffee Intake and Common Mental Disorders: Insights From Genetic Analysis

**DOI:** 10.1111/cns.70213

**Published:** 2025-01-07

**Authors:** Zhiqiang Du, Ying Jiang, Yuan Shen, Qin Zhou, Peng Gong, Haohao Zhu

**Affiliations:** ^1^ Affiliated Mental Health Center of Jiangnan University Wuxi Central Rehabilitation Hospital Wuxi China

**Keywords:** Coffee intake, Mendelian randomization, Mental disorders, Obsessive‐compulsive disorder

## Abstract

The study found a significant causal relationship between coffee intake and obsessive‐compulsive disorder, showing a negative correlation. There was no causal relationship between coffee intake and other mental disorders. The sensitivity analysis test found no pleiotropy affecting the results, and no single nucleotide polymorphism had a major impact on the robustness of the results, indicating that the results are stable and reliable.

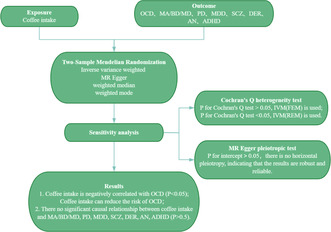

The significance of mental disorders cannot be overstated, as they impact over 970 million individuals globally, accounting for approximately 14.39% of disability‐adjusted life years [1]. These disorders, including obsessive‐compulsive disorder (OCD), major depressive disorder (MDD), bipolar disorder (BD), schizophrenia (SCZ), and attention‐deficit/hyperactivity disorder (ADHD), profoundly affect individuals' cognitive, emotional, and behavioral functioning.

Our study aimed to elucidate the potential causal association between coffee intake and the risk of mental disorders through a two‐sample Mendelian randomization approach. Utilizing extensive genome‐wide association study (GWAS) data from the European population, we selected single nucleotide polymorphisms associated with coffee consumption as instrumental variables (IVs). By analyzing these IVs, we sought to minimize confounding factors and ascertain whether coffee intake can influence the onset of mental health issues.

The findings of our study revealed a significant negative correlation between coffee intake and the risk of developing OCD, evidenced by an odds ratio of 0.333 (95% CI: 0.117–0.943, *p* = 0.038). This suggests that higher coffee consumption may reduce the likelihood of OCD symptoms developing in individuals predisposed to this disorder. Although the association with OCD is noteworthy, it is notable that our analysis did not find a significant potential causal association between coffee intake and other mental disorders, such as MDD, BD, PD, SCZ, or ADHD (*p* > 0.05; Figure 1). This specificity in our findings emphasizes the potential unique role of caffeine in mitigating OCD‐related behaviors and cognitive patterns.

**FIGURE 1:**
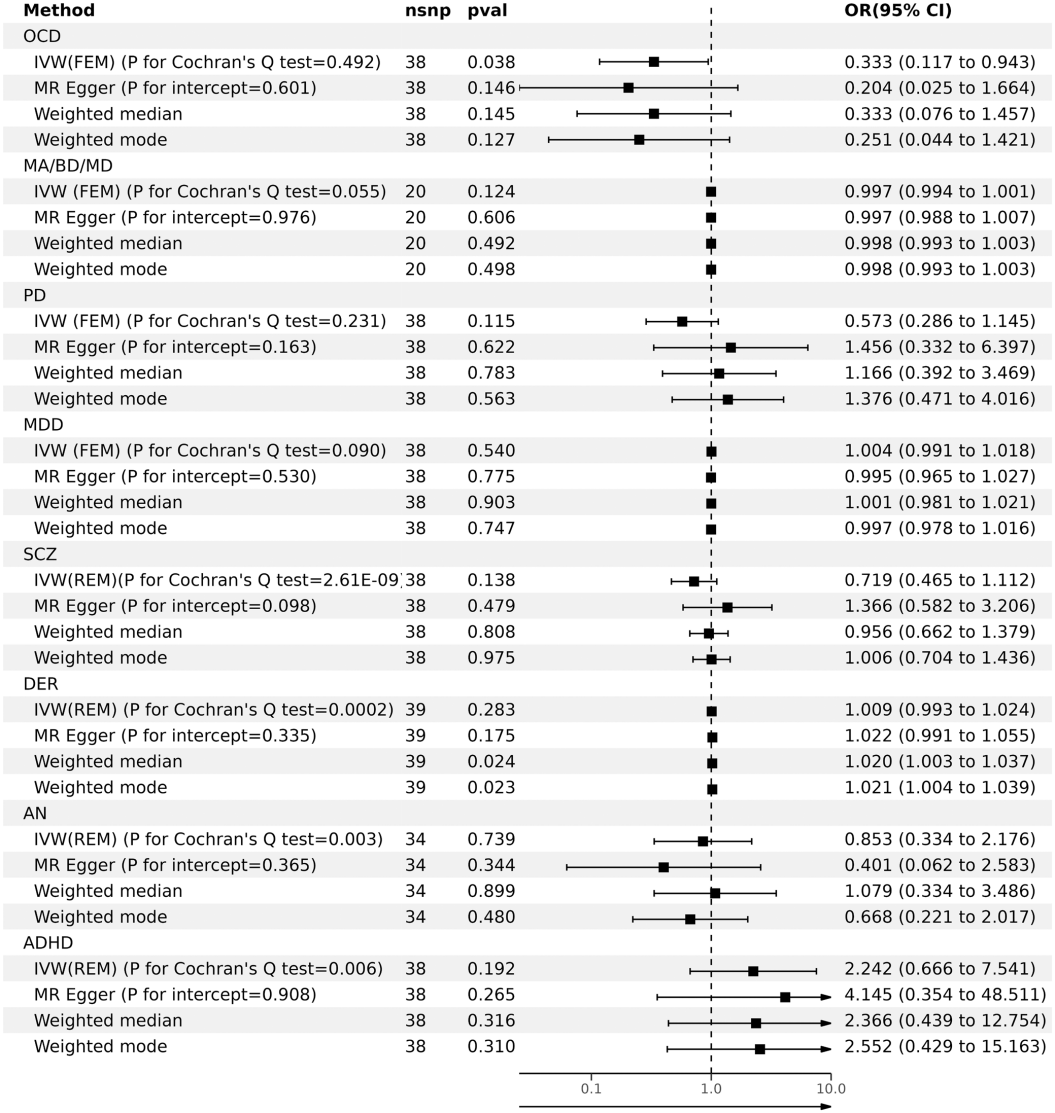
Potential causal association between coffee intake and eight common mental disorders. 95% CI, 95% confidence interval; ADHD, attention‐deficit/hyperactivity disorder; AN, anorexia nervosa; DER, depression; IVW, inverse variance weighted; MA/BD/MD, mania/bipolar disorder/manic depression; MDD, major depressive disorder; nsnp, number of single nucleotide polymorphisms; OCD, obsessive‐compulsive disorder; OR, odds ratio; PD, panic disorder; SCZ: schizophrenia. *p* < 0.05 indicates statistical significance. If *p* for Cochran's *Q* test > 0.05, the fixed effects model (FEM) within the IVW framework is applied; if *p* for Cochran's *Q* test < 0.05, the random‐effects model (REM) is used. A *p*‐value for the intercept > 0.05 indicates no horizontal pleiotropy, suggesting that the results are robust and reliable.

The biological mechanisms underlying this relationship may be linked to caffeine's effects on the central nervous system. Caffeine is known to enhance alertness, promote the release of neurotransmitters like serotonin and dopamine, and modulate brain activity. These effects could play a crucial role in improving cognitive control and reducing the impulsivity characteristic of OCD [2, 3]. Previous research indicates that caffeine consumption may diminish distress responses to anxiety‐provoking stimuli, thereby potentially aiding individuals in managing their compulsive behaviors [4]. The mechanism of action of caffeine primarily involves antagonism of adenosine A1 and A2 receptors. The A1 receptor regulates the release of various neurotransmitters, including tryptophan, serotonin, and norepinephrine; its inhibition can lead to changes in the release and reuptake of these neurotransmitters, which are associated with the pathophysiology of OCD [5].

Moreover, the implications of our research extend beyond OCD. Understanding how coffee consumption affects various mental health outcomes could inform dietary recommendations and therapeutic strategies for individuals suffering from these disorders. As coffee is one of the most widely consumed beverages globally, the potential for dietary interventions to contribute to mental health management is significant.

Despite the strengths of our study, including the utilization of robust genetic data and a comprehensive analytical framework, we acknowledge certain limitations. The generalizability of our findings may be constrained by the fact that the GWAS data primarily reflect European populations. Further research is essential to explore whether these findings hold true across diverse ethnic groups and varying demographic factors, including gender. Additionally, although our study focused on established mental disorders, future investigations could delve into subclinical symptoms and broader psychosocial factors that may also be influenced by coffee consumption.

This research contributes to the burgeoning literature on the impact of dietary factors on mental health, particularly regarding the role of coffee. Previous studies have yielded mixed results, with some suggesting that coffee intake can exert a protective effect against depression, whereas others indicate potential adverse effects on mood in certain populations. Our findings provide a clearer perspective on this contentious issue, highlighting the need for nuanced discussions about the role of caffeine in mental health management.

We believe that our study fills a critical gap in the current understanding of coffee intake's impact on mental disorders and provides an important contribution to future clinical research. The findings advocate for further investigation into the therapeutic potential of coffee and its constituents in managing mental health conditions, particularly OCD. Given the rising prevalence of mental disorders and the ongoing search for effective prevention strategies, our research offers new insights that could inform public health initiatives and clinical practices.

## Author Contributions

**Zhiqiang Du and Haohao Zhu:** conceived the study. **Ying Jiang, Qin Zhou, Yuan Shen, and Peng Gong:** collected the report. **Zhiqiang Du and Haohao Zhu:** wrote the manuscript and edited the manuscript. All authors have approved publishment of the manuscript.

## Ethics Statement

The authors have nothing to report.

## Consent

The authors have nothing to report.

## Conflicts of Interest

The authors declare no conflicts of interest.

## Data Availability

The dataset generated during and analyzed during the current study is available from the MR Base database (http://www.mrbase.org/).
